# Eudragit-Coated Sporopollenin Exine Microcapsules (SEMC) of *Phoenix dactylifera* L. of 5-Fluorouracil for Colon-Specific Drug Delivery

**DOI:** 10.3390/pharmaceutics13111921

**Published:** 2021-11-12

**Authors:** Mohammad Raish, Mohd Abul Kalam, Ajaz Ahmad, Mudassar Shahid, Mushtaq Ahmad Ansari, Abdul Ahad, Raisuddin Ali, Yousef A. Bin Jardan, Aws Alshamsan, Musaed Alkholief, Khalid M. Alkharfy, Ibrahim Abdelsalam Abdelrahman, Fahad I. Al-Jenoobi

**Affiliations:** 1 Department of Pharmaceutics, College of Pharmacy, King Saud University, P.O. Box 2457, Riyadh 11451, Saudi Arabia; makalam@ksu.edu.sa (M.A.K.); mahmad@ksu.edu.sa (M.S.); aahad@ksu.edu.sa (A.A.); ramohammad@ksu.edu.sa (R.A.); ybinjardan@ksu.edu.sa (Y.A.B.J.); aalshamsan@ksu.edu.sa (A.A.); malkholief@ksu.edu.sa (M.A.); ph.ibrahimabdalsalam@gmail.com (I.A.A.); aljenobi@ksu.edu.sa (F.I.A.-J.); 2 Nanobiotechnolgy Unit, Department of Pharmaceutics, College of Pharmacy, King Saud University, P.O. Box 2457, Riyadh 11451, Saudi Arabia; 3 Department of Clinical Pharmacy, College of Pharmacy, King Saud University, P.O. Box 2457, Riyadh 11451, Saudi Arabia; aajaz@ksu.edu.sa (A.A.); alkharfy@ksu.edu.sa (K.M.A.); 4 Department of Pharmacology and Toxicology, College of Pharmacy, King Saud University, P.O. Box 2457, Riyadh 11451, Saudi Arabia; muansari@ksu.edu.sa

**Keywords:** 5-fluorouracil, pollen, *Phoenix dactylifera*, Eudragit^®^-RS100, coating, colon-specific

## Abstract

In this study, 5-fluorouracil (5-FU)-loaded pollens of Phoenix dactylifera and their coating with ERS was done and evaluated for the colon-targeted delivery of 5-FU to treat colon cancer. Sporopollenin exine microcapsules (SEMC) from the pollens of Phoenix dactylifera were extracted by the reflux method and 5-FU into SEMC was encapsulated by the vacuum-assisted loading method. 5-FU loaded SEMC was coated with Eudragit^®^ RS-100 (ERS) by the organic solvent-evaporation technique under vacuum to avoid the discharge of 5-FU in the stomach and small intestine. Morphological and physicochemical characterization of drug-loaded SEMC (coated/uncoated) was performed by scanning electron microscopy (SEM), FTIR, XRD, and DSC. The encapsulation and drug loading were determined by the direct method, and an in vitro release study was performed in simulated gastric and intestinal fluids (SGF/SIF). The colon-specific delivery of 5-FU from the SEMC was assessed in terms of pharmacokinetics and gastrointestinal tract distribution after oral administration in rats. The successful encapsulation and loading of 5-FU into SEMC by a vacuum-assisted loading technique and its coating with ERS by a solvent-evaporation technique were achieved. SEM images of uncoated SEMC have shown porous structures, and coating with ERS reserved their morphology with a smooth surface and discrete microstructures and the 5% *w/v* ERS acetone solution. ERS-coated SEMC sustained the release of 5-FU until 24 h in SIF, while it was up to 12 h only from uncoated SEMC. The maximum plasma concentration (Cmax) of 5-FU from uncoated SEMC was 102.82 μg/mL after 1 h, indicating a rapid release of 5-FU in the upper gastrointestinal tract. This concentration decreased quickly with a half-life of 4 h, AUC0-t was 264.1 μg/mL.h, and MRT0-inf was 5.2 h. The Cmax of 5-FU from ERS-coated SEMC was 19.47 μg/mL at 16 h. The Cmax of 5-FU in small intestines was 406.2 μg/g at 1 h from uncoated SEMC and 1271.5 μg/g at 12 h from coated SEMC. Conclusively, a 249.9-fold higher relative bioavailability of 5-FU was achieved with the ERS-coated SEMC in colon tissues than that from uncoated SEMC.

## 1. Introduction

5-Fluorouracil (5-FU) is commonly used for chemotherapy to treat various solid tumors of colorectal, breast, stomach, intestine, and colon metastatic carcinomas [1,2,3,4]. 5-FU infusions are the common mode of treatment; however, infusions are often troublesome, expensive, and repetitive, and high doses are required due to the short half-life of 5-FU (15 to 20 min) [5]. Furthermore, 5-FU is absorbed via blood capillaries in systemic circulation, leading to a reduction in drug level at the tumor site, loss of anticancer efficacy, and systemic toxicity in the clinic due to multidrug resistance The development of controlled oral delivery of 5-FU formulations would be vastly advantageous; several overtures to develop controlled release oral formulations of 5-FU have been described for colon targeting [6,7,8]. The multiparticulate drug delivery system (MDDS) is widely used due to the dose distribution to small subunits, and release of the drug can be customized to therapeutic requirements [9,10]. Controlled-release MDDS for 5-FU has been reported by encapsulating the 5-FU into various polymeric microspheres, but these are highly expensive [7,11]. Plants spores (phoenix dactylifera) and pollen grains as MDDS have been used due to high structural uniformity, porosity, adsorptive surface, and micro-scale size distribution [12,13]. Phoenix dactylifera spores are economical and can be easily processed on an industrial scale. The combination therapy is extensively used for the management of cancer to circumvent the poor response of chemotherapy and multidrug resistance [14]. Several reports established that co-encapsulation enhances the efficacy of chemotherapy and reduced the multidrug resistance of chemotherapy using MDDS [15,16,17,18]. Sporopollenin consists of natural microcapsules obtained from pollen grains of plants and is a highly stable natural biocompatible biopolymer that has an edge over synthetic natural polymers. Sporopollenin exine microcapsules (SEMC) have a large internal cavity consisting of interlinked pores of uniform size. Such uniformity is tough and costly to attain by man-made material, and the microcapsules are capable of encapsulating a wide range of polar and non-polar drugs [19,20,21]. The size, structure, surface properties, and porous morphology make them suitable for controlled drug release, loading, and delivery vehicle [12,22]. The combination of sinapic acid (SA) and 5-FU exhibit a synergetic anticancer effect with minimal side effects [23]. Piperine is a chemopreventive agent that inhibits P-glycoprotein and/or CYP3A4; therefore, it increases the bioavailability of drugs that are the substrate for PGP/CYP3A as well as increasing the sensitivity of cancer cells at the site of tumors [24]. The inhibitory effect on PGP/CYP3A can enhance the sensitivity, reverse multidrug resistance, and act as a bioavailability-increasing agent for several chemotherapeutic agents [24,25]. Herein, we report the encapsulation of 5-FU into SEMC obtained from Phoenix dactylifera by the vacuum-loading technique and their coating with Eudragit^®^-RS100 (E-RS) for the colon-targeted delivery of 5-FU. A colon-specific delivery system has great potential to deliver many therapeutic agents, proteins, and peptides to treat the local colonic ailments because of the less aggressive environmental condition of the colon to deliver the drugs [26]. The less aggressive environmental condition of the colon exerts less multiplicity and intensity of enzymatic actions at the near-neutral pH of the colon [26,27,28,29]. Eudragit-RS100 (E-RS) is a copolymer of ethyl acrylate, methylmethacrylate, and methacrylic acid esterified with quaternary ammonium groups [30]. It is used as a coating material for a pH-dependent colon-targeted oral drug delivery system [31]. Due to their unique physicochemical characteristics and diverse usage, Eudragits have been employed to develop enteric-coated, sustained-release, and colon-specific drug delivery carriers [32]. Moreover, ERS is less hydrophilic than E-RL; hence, the slow release of most of the drugs is expected when E-RS is used for coating purposes [26]. Thus, we have chosen ERS as a coating material for 5-FU-loaded SEMC to achieve prolonged release of 5-FU in the colonic environment.

## 2. Materials and Methods

### 2.1. Materials

*Phoenix dactylifera* L. (date palm) pollens were procured from indigenous farms in March from Riyadh, Saudi Arabia. Eudragit^®^ RS-100 (C_19_H_34_ClNO_6_), mol wt. 32,000 g/mol was a kind gift of Evonik Corporation (Former Evonik Degussa GmbH). 5-Fluoro-2,4 (1H, 3H)-pyrimidinedione (C_4_H_3_FN_2_O_2_), ammonium hydroxide solution (NH_4_OH), and ethanol were purchased from Sigma-Aldrich (St. Louis, MO, USA). Phosphate buffer (pH 6.8) was prepared as per the European Pharmacopoeia. Water was obtained by Milli-Q^®^ water purifier (Millipore, Paris, France). All other used chemicals were of analytical grade, and the solvents were of HPLC grade.

### 2.2. Methods

#### 2.2.1. Pollen Collection

The obtained *Phoenix dactylifera* (date palm) pollens were passed through a sieve using 10–50 µm, and the identification of the pollens was confirmed by light microscopy. Pollen grains were stored at −20 °C in sealed glass bottles. *Phoenix dactylifera* pollen is monocolpate, symmetric, oval-elliptic, and oblong; mean values of the polar axis and equatorial diameter of the pollen grains were measured as 15–26 μm and 22–25 μm, respectively.

#### 2.2.2. Extraction of Sporopollenin Exine Microcapsules (SEMC)

The extraction of sporopollenin exine microcapsules was performed by a reported method [33]. Briefly, 50 g of *Phoenix dactylifera* (date palm) pollens were treated with 150 mL of acetone under reflux for 6 h. The defatted spore powder (DFS) was obtained after filtrations [33,34]. DFS was treated with 10% acetic acid and then with 10% sodium hydroxide followed by washing in hot water to get hydrolyzed sporopollenin powder (HSP) [35]. The HSP was treated with 15% potassium hydroxide and refluxed for 10 h. Then, we filtered the solution followed by washing with hot water and ethanol several times to get base hydrolyzed sporopollenin (BHS). The dried BHS was treated with 100 mL of orthophosphoric acid and refluxed with stirring for 72 h. The resulting mixer was filtered and washed several times with water, acetone, and ethanol and filtered dried at 60 °C to get SEMC [12].

#### 2.2.3. Encapsulation of 5-FU into SEMC

The encapsulation of 5-FU into SEMC was performed by a vacuum-assisted loading technique [22]. Accurately weighed amounts (50, 100, and 150 mg) of 5-FU were dissolved in 2 mL of 1:1 (*v*/*v*) mixture of 1N NH_4_OH: ethanol. Around 200 mg of SEMC was suspended into the 5-FU solution. The obtained suspension was vortexed for 5 min. Then, the suspension was placed in a freeze dryer (FreeZone 4.5 Freeze Dry System, Labconco Corporation, Kansas City, MO, USA) at −20 °C temperature and 1 mBar vacuum. After 3 h, freeze drying was stopped and 5-FU-loaded SEMC was washed thrice with Milli-Q water (5 mL in each cycle) by centrifugation (at 15,000 rpm for 15 min at 4 °C by using ultracentrifuge (PRISM-R, Labnet International Inc. Edison, NJ, USA) to remove the unbounded or surface-bonded 5-FU. The SEMC was kept at −80 °C for 3 h and lyophilized for 24 h (coded as 5-FU-SEMC). The drug-containing SEMC was further coated with ERS, and so the obtained spores (SEMC) were stored at the dry place in Falcon tubes for further characterization.

#### 2.2.4. Formulation of Eudragit^®^ RS-100 (E-RS) Coated SEMC

The optimized drug-loaded spores were coated by 2.5%, 5%, and 10%, *w/v* organic solution of E-RS. The coating solution was prepared by dissolving ERS in 5 mL of acetone [22]. For the coating process, 100 mg of drug-loaded SEMC were added to the 5 mL of ERS solution. The dried SEMC was obtained by evaporating the acetone under vacuum at 40 °C for 2 h using a rotary evaporator (Buchi™ Rotavapor^®^ 210, Switzerland). Then, the obtained dried SEMC were gently powdered using a mortar and pestle and stored in Falcon tubes at the dry place for further studies.

#### 2.2.5. Morphological Characterization of SEMC and Size Analysis

The morphological characterization of ERS-coated (F2-ERS) and uncoated (F2) drug-loaded SEMC was performed by scanning electron microscopy (SEM) (Zeiss EVO LS10; Cambridge, UK) using the gold sputter technique. The products were coated with gold in the “Q150R Sputter unit” from Quorum Technologies Ltd. (East Sussex, UK) in an argon atmosphere at 20 mA current for 2 min. Scanning electron microscopy was performed at an accelerating voltage of 15 kV, 6.5–7 mm working distance, and at varying 5000, 10,000, and 15,000 times of magnification. Binning analysis was performed to investigate the average size of the SEMC using the software ImageJ (V-1.53a, National Institutes of Health, Bethesda, MD, USA). The size distributions of the SEMC were investigated by utilizing the size data obtained during the binning analysis (by ImageJ) through the “ORIGIN^®^ 8.5 Data Analysis and Graphing Software” (OriginLab Corporation, Northampton, MA, USA).

#### 2.2.6. Porosity Determination of SEMC

The specific surface volumes, porosity, and pore size distribution of the ERS-coated SEMC-FU (F2-ERS) and uncoated SEMC-FU (F2) and SEMC alone were determined by nitrogen (N2) adsorption–desorption isotherms measurements following the reported method [13]. The measurements were performed on a TriStar-3000 Instrument (Micrometrics Inc., Norcross, GA, USA).

#### 2.2.7. FTIR Spectra

The FTIR spectra of pure drug 5 FU, Eudragit RS-100, macroporous SEMC, drug-loaded uncoated SEMC-FU (F2), and the ERS-coated SEMC-FU (F2-ERS) were recorded in the range of 4000–450 cm^−1^ by a Bruker Tensor-27 spectrophotometer (USA) using KBr pellets.

#### 2.2.8. Powder X-ray Diffraction

The powder X-ray diffractions of the samples were carried out by Ultima-IV Goniometer (Rigaku, Inc., Tokyo, Japan) over the 2*θ* (deg) range from 3.0 to 70.0 deg at 1.0 deg/min of scan speed to examine the crystalline nature of the samples (5-FU-loaded SEMC, 5-FU-loaded and ERS-coated SEMC in comparison to the pure 5-FU). The X-ray tube (anode material) was Cu with Ka2 elimination, where the Ka2/Ka1 intensity ratio was 0.10 nm, and it was monochromatized with the graphite crystal. The diffractograms were obtained at 40 kV of tube voltage and 40 mA of the generator with the given specifications (DivSlit: 1/2 deg, DivH.L. Slit: 10 mm SctSlit: 1/2 deg and RecSlit: 0.3 mm) where the step scan mode of step size 0.02° and counting time was 1 sec per step.

#### 2.2.9. Differential Scanning Calorimetry

Thermal analysis of pure drug 5-FU, Eudragit RS-100, macroporous SEMC, drug-loaded uncoated SEMC-FU (F2), and the ERS-coated SEMC-FU (F2-ERS) were conducted using a differential scanning calorimeter (Netzsch, DSC 200F3, Selb, Germany). The sample cells were purged by nitrogen at a flow rate of 50 mL/min. An aliquot of approximately 5 mg was weighed and sealed in an aluminum pan, and an empty pan was used as a reference. The thermal behaviors of all samples were scanned from −10 to 240 °C at a heating rate of 10 K/min.

### 2.3. Chromatographic Analysis of 5-FU

The HPLC-UV method was used for the routine analysis of 5-FU. Previously reported HPLC-UV methods [36,37,38] were used to analyze 5-FU in the samples obtained from encapsulation, drug loading, and in vitro drug release experiments. The HPLC system (Waters-1500 series controller, USA), comprised of a UV-detector (Waters-2489, dual absorbance detector), binary pump (Waters-1525), and an automated sampling system (Waters-2707 plus autosampler) was used for the assay of 5-FU. The HPLC system was controlled and monitored by “Breeze-software”. 5-FU was analyzed by injecting 30 μL of the supernatant into the column (MACHERY-NAGEL, EC150/4.6 NUCLEODUR C18, Gravity, 5 μm) maintained at 30 °C (38). The mobile phase (40 mM KH2PO4 buffer, pH was adjusted to 7 by 2%, *w/v* KOH) was pumped with a flow rate of 1 mL·min^−1^. The volume of injection was 30 μL, the run-time was 10 min, and UV detection of 5-FU was done at 260 nm [39].

The standard stock solution of 5-FU (1000 µg·mL^−1^) was prepared in methanol; from this solution, 0.25–100 µg·mL^−1^ concentration ranges were prepared by serial dilutions with the mobile phase. The calibration curve was obtained by plotting the known concentrations of 5-FU (µg·mL^−1^) versus the corresponding peak area. The calibration curve was linear in the mentioned concentration range with the 0.9999 value of coefficient of determinations (R^2^). The obtained regressed equation was successfully employed for the quantitative analysis of 5-FU during encapsulation, drug loading, and in vitro drug release experiments. The same HPLC-UV method was used to analyze 5-FU in plasma samples and tissue homogenates with slight modification [40]. The standard stock solution of 5-FU (1000 µg·mL^−1^) was prepared in methanol. The stock solution of thymine as internal standard (IS) was prepared in methanol at 1000 µg·mL^−1^ concentration [41]. The calibration curve was prepared by spiking 5-FU solution into 400 µL of rat plasma to get 0.25–100 µg·mL^−1^ concentration ranges; then, 50 µL of IS (50 µg) was added to each sample. Acetonitrile (250 µL) was added to the spiked samples for protein precipitation. Then, a 2 mL mixture of isopropanol and diethyl ether (16: 84, *v/v*) was added to each sample, vortexed for 30 s, and centrifuged at 5000 rpm for 5 min. The organic layer was dried under a nitrogen stream, the obtained residue was dissolved in 1000 µL of mobile phase, and 30 µL of this sample was injected into the HPLC system for the analysis [42]. The peaks of 5-FU and IS appeared separately at the retention times of 4.25 and 6.35 min, respectively. Similarly, tissue homogenates 400 µL (0.1 g tissue/mL) were transferred to 1.5 mL Eppendorf tubes. The samples were spiked with 50 μL of IS (50 μg) and mixed by vortexing. Afterward, the rest of the process was followed as mentioned for plasma samples.

### 2.4. Encapsulation Efficiency (%EE) and Drug Loading (%DL)

The encapsulation of 5-FU into SEMC was determined by the direct method. For this, 10 mg of 5-FU-loaded SEMC were suspended in 10 mL of phosphate-buffered saline (PBS, pH 7.4) and vortexed for 5 min; then, the mixture was pulse sonicated by probe sonication (Sonics & Materials, Inc. Newtown, CT, USA) at 40% power for 45 sec on ice bath (3 cycles 15 s each). The suspension was centrifuged (at 6000 rpm for 5 min), the supernatant was collected, and the concentration of 5-FU was measured by the HPLC-UV method as described above. The encapsulation efficiency (%EE) and drug loading (%DL) were calculated by the following equations:

(1) $$\%EE=\left(\;\frac{Amount\;of\;drug\;loaded/determined\;\left(mg\right)}{Initial\;amount\;of\;drug\;\left(mg\right)}\right)\times 100\;$$

(2) $$\%DL=\left(\;\frac{Amount\;of\;drug\;loaded/determined\;\left(mg\right)}{Total\;amount\;of\;drug\;loaded\;SEMC\;\left(mg\right)}\right)\times 100$$

### 2.5. In Vitro Drug Release Study

In vitro release of 5-FU was performed in aqueous HCl solution as simulated gastric fluid (SGF, pH 1.2) for 2 h followed by phosphate buffer solution (PBS, pH 6.8) to simulate the conditions of gastrointestinal tract up to 36 h. Accurately weighed 10 mg of drug-loaded SEMC (both the uncoated and E-RS coated) were added in release media (50 mL) in 100 mL capacity beakers and allowed to be shaken (at 50 rpm) in a shaking water bath maintained at 37 ± 0.5 °C. At different time intervals, 1 mL of aliquots was taken out from the beakers, and the same volume of fresh release media was put into the beakers to maintain the sink condition. The obtained samples were centrifuged at 5000 rpm for 5 min. The supernatant was collected, and the drug concentrations were measured by the HPLC-UV method as described above. Both the coated and uncoated formulations were used in triplicate for this experiment.

### 2.6. Stability of 5-FU-Loaded Uncoated and ERS-Coated SEMC

A short-term stability of 5-FU-loaded SEMC (F2 and F2-ERS) was conducted by following the methods [43,44]. Approximately 10 mg of 5-FU-loaded freeze-dried SEMC (F2 and F2-ERS) were packed separately into tightly closed glass containers and stored at 30 ± 1 °C for 30 days (as per the climatic zone of Saudi Arabia (IVa)). Time-dependent changes in the size, %EE, and %DL were determined on the 15th and 30th days to realize the stability of the optimized F2 and ERS coated F2.

### 2.7. In Vivo Study

#### 2.7.1. Animals

Male Wister rats (≈12 weeks old) weighing 185–203 g were acquired from the Central Animal House Facility of King Saud University. The rats were kept in the cages with 12 h light and dark cycle at 25 ± 2 ºC. The animals were fed on standard rat chow and provided water ad libitum. The Research Ethics Committee of College of Pharmacy, King Saud University approved the study (Ethical Reference No: KSU-SE-21–59). All animals used in the experiments received care in compliance with the NIH Guideline for the Care and Use of Laboratory Animals.

#### 2.7.2. Pharmacokinetics and Gastrointestinal Distribution Study

The efficiency of 5-FU-loaded SEMC for the colon-specific delivery of the drug was evaluated for the pharmacokinetic and GI-tract distribution in rats. Animals were fasted overnight before the experiments, but water was provided ad libitum during the experiments. The animals were divided into two groups (Group I and Group II) each consisting of 33 animals. The animals of Group I and Group II were given an equivalent amount of coated SEMC (F2-ERS) and uncoated SEMC (F2), respectively, each containing 8.05 mg of 5-FU by oral gavage. The administered dose of 5-FU was calculated according to the following Equation (3), as reported previously [45,46].

(3) $$Surface\;area=\frac{\mathrm{Colon}\;\mathrm{area}\;\left({\mathrm{cm}}^{2}\right)\times Dose\;\left(500\;\mathrm{mg}\cdot m^{-2}\right)\times \mathrm{Km}\;rat\;for\;250\;g}{\mathrm{Colon}\;\mathrm{length}\;\left(\mathrm{cm}\right)}$$

The calculated dose was found to be 8.05 mg. After dosing, three rats from each group were euthanized at predetermined time points by carbon dioxide (CO_2_) inhalation. Around 3 mL of blood samples were collected by cardiac puncture into heparinized vacutainers and centrifuged at 5000 rpm for 10 min; then, plasma was collected and stored at −20 °C until the analysis of 5-FU was performed by UPLC-UV. Directly after euthanization, rats were placed on ice packs and opened by bilateral thoracotomy. The full GI tract was detached, and the mesenteric and fatty tissues were separated. The GI tract was segmented into the stomach, small intestine, caecum, and colon. The contents of the lumen were removed by gentle pressure with wet scissors, and organs were cut longitudinally and washed with normal saline to remove the remaining luminal contents. The colon was weighed and cut into small pieces and homogenized at 4 °C with an Ultra-turrex (type T 25) homogenizer (IKA-Werke, Staufen, Germany). Then, the homogenate was centrifuged at 5000 rpm for 10 min at 4 °C. The fatty layer was discarded, and the amount of 5-FU in the supernatant was quantified by HPLC-UV. The pharmacokinetic data were analyzed by fitting to a non-compartmental model using PK-Solver, V-1.0 [47].

### 2.8. Statistical Analysis

Statistical analysis was performed using one-way analysis of variance (ANOVA) with a Kruskal–Wallis comparisons test for non-parametric data. The *p*-value < 0.05 was considered as statistically significant. The encapsulation of 5-FU with natural spores and in vitro release experiments was performed in triplicate, and all the data were expressed as mean ± SD, *n* = 3.

## 3. Results and Discussion

### 3.1. Formulation of 5-FU-Loaded SEMC and Its Coating by ERS

We tried varying amounts of 5-FU (50, 100, and 150 mg) to encapsulate and load into the SEMC by keeping a constant amount of SEMC (200 mg) in each case (Table 1). To improve the encapsulation of 5-FU into SEMC, initially, an increased amount of 5-FU was solubilized in a 1:1 (*v*/*v*) mixture of NH_4_OH: Ethanol. SEMC were suspended into the hydro-alcoholic solution of 5-FU and subjected to vacuum-assisted (at −20 °C and 1 mBar) drug loading, which causes the entrance of the drug into the internal cavities of the spores through the nanoscale channels present on the surfaces of SEMC [48]. The use of a higher amount of drug did not facilitate the highest amount of drug encapsulation and loading; rather, an optimum amount (100 mg) of 5-FU has shown a sufficient encapsulation and loading into SEMC, which might be due to the saturation solubility of the drug into the used aqueous phase [49]. Employing ammonia (NH_4_OH) could prevent possible complications that might occur during the use of amines. Since ammonia is a small molecule, it could provide maximum access to the available functional groups on SEMC. Moreover, ammonia is highly water soluble and volatile, so any unreacted ammonia would not contaminate the 5-FU-loaded SEMC, which was also reported in previous studies [12,22,50]. Successful encapsulation and loading, to prevent the oxidation of proteins [20] and enzymes [33] into *Phoenix dactylifera* spores, were obtained by a vacuum-assisted technique. Therefore, the loading and optimization of 5-FU into SEMC in the present study by a vacuum-assisted technique offered superior acumen to obtain an improved encapsulation of low aqueous soluble drug. Of the varying concentrations (2.5%, 5%, and 10%, *w*/*v*) of ERS organic solution, 5% solution was found to be best for the surface coating of 5-FU-loaded SEMC, as the Scanning Electron Microscope (SEM) image of the coated SEMC has shown a discrete structure with smooth surfaces.

### 3.2. Structural Morphology and Size Analysis of SEMC

**Figure 1:**
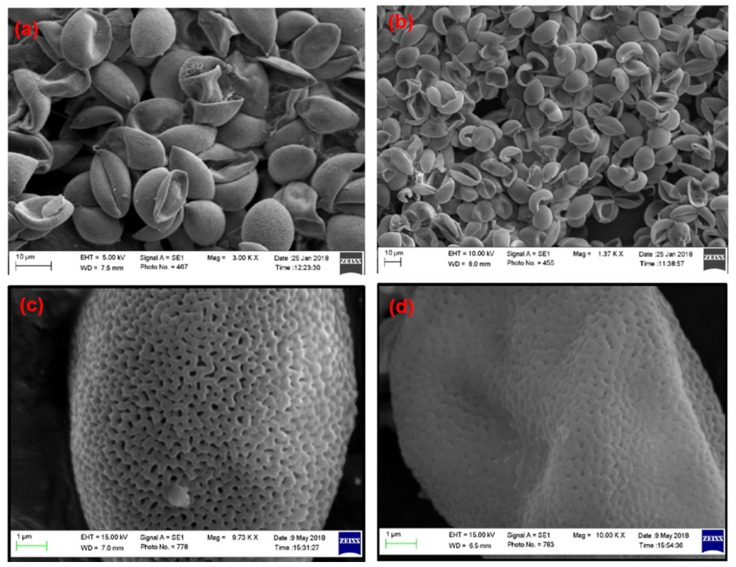
SEM images of 5-FU-loaded SEMC: (**a**,**c**) are the images for F2-uncoated while (**b**,**d**) are for Eudragit^®^ RS-100-coated F2.

The morphological structure of 5-FU-loaded SEMC before and after ERS coating was evaluated by SEM. The scanned images are represented in Figure 1. Figure 1a,b represent the SEM images of the uncoated SEMC and ERS-coated SEMC, respectively, at low magnifications and large scale (10 µ), while Figure 1c and d are the SEM images of the uncoated SEMC and ERS-coated SEMC, respectively at higher magnifications and small scale (1 µ). The surface of SEMC before coating (Figure 1c) clearly shows prominent reticular and porous structures as previously reported [22,51]. In the case of coating with ERS, the microstructure of the spores remained unchanged (Figure 1d) and reserved its morphology [49]. There were no damaging effects or cracking observed even after the application of external factors such as the application of vacuum at −20 °C and evaporation of organic solvent at 40 °C for ERS coating [22].

**Figure 2:**
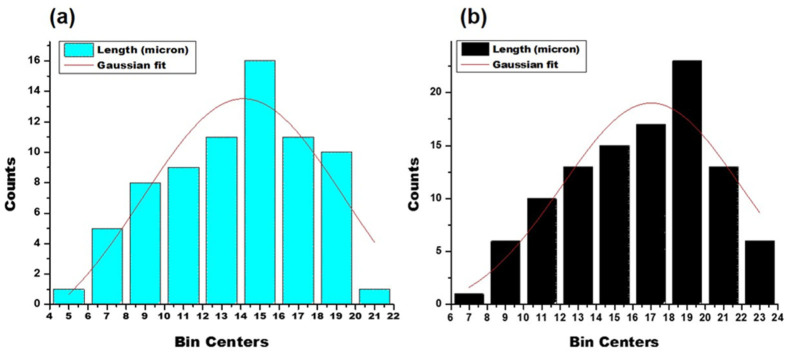
Particle size distribution plots of 5-FU-loaded SEMC: uncoated (**a**); ERS coated (**b**).

The surface of the ERS-coated and 5-FU-loaded SEMC was smooth and discrete, indicating that the microchannels were partially covered due to the polymer coating. Thus, we conclude that the SEM images substantiate that the method of polymer coating provided a well-defined surface structure with a uniform size distribution of the spores. Moreover, the smoothness of the surface of drug-loaded ERS-coated SEMC indicated that the 5-FU were predominantly encapsulated into the internal cavities of the SEMC [52]. The average sizes of the 5-FU-loaded SEMC (F1–F3) are summarized in Table 1. The sizes of uncoated SEMC (F2) and ERS-coated (F2-ERS) were found in the range of 4.89–20.28 μm and 8.14–23.71 μm, respectively using ImageJ analysis. The size distribution plots of the uncoated (F2) and polymer-coated F2 are represented in Figure 2. The size distribution analysis of the optimized formulations (F2 and F2-ERS) demonstrated a direct relationship between the polymer concentration and size of the SEMC, which could also be observed visually in the SEM images (Figure 2).

### 3.3. Porosity and Surface Volume Measurement

**Figure 3:**
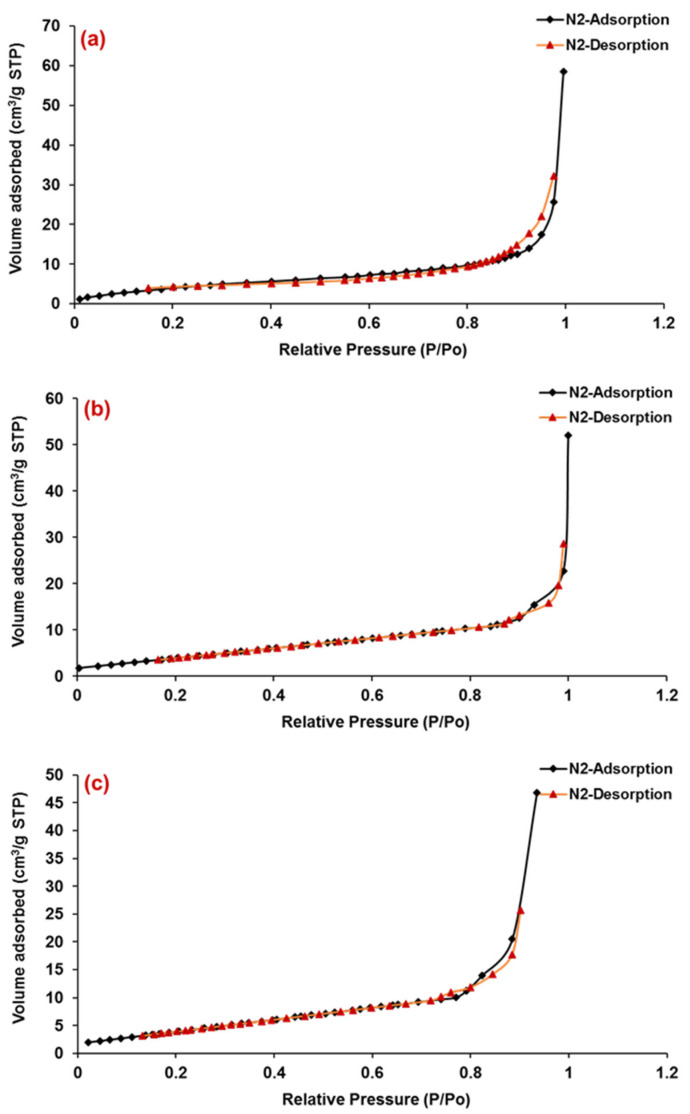
Nitrogen (N_2_) adsorption–desorption isotherm of macroporous SEMC (**a**), 5-FU-loaded SEMC (F2) (**b**) and ERS-coated 5-FU-loaded SEMC (F2-ERS) (**c**). All measurements were performed at 77 K.

The adsorption analysis by N_2_ gas is frequently used for the measurement of porosity and surface area. Here, we exposed the porous and solid SEMC to N_2_ at liquid nitrogen conditions (i.e., 77 K) as probe molecule at different conditions and evaluated the weight uptake/the volume of N_2_ adsorbed by macroporous SEMC, 5-FU-loaded SEMC (F2) and the ERS-coated SEMC (F2-ERS). Here, the software of the instrument (TriStar-3000) employed the BET technique/equation to the portion of the isotherm to examine the surface area of the porous SEMC [53]. The pore size distribution of the macroporous SEMC, 5-FU-loaded SEMC (F2), and the ERS-coated (F2-ERS) was examined using Langmuir N_2_ adsorption–desorption isotherms, as illustrated in Figure 3. Macroporous SEMC, drug-loaded SEMC (F2), and (F2-ERS) displayed a type-II adsorption–desorption isotherm, corresponding to the macrospore materials [54]. In the cases of F2 and F2-ERS, the adsorbed amount of N_2_ was reduced, but the shape of the hysteresis loop remained similar. The surface pore volume and average pore diameter of SEMC were 90.72 cm^3^/g and 27.84 Å, respectively. After being adsorbed with FU and coated with ERS, these parameters were further reduced to 71.63 cm^3^/g and 24.86 Å for F2 and 50.54 cm^3^/g and 22.09 Å for F2-ERS, respectively. These results revealed that after polymer coating on the surface of SEMC, the pore shape did not change significantly because the polymer distributed predominantly on the exterior surface of SEMC, suggesting that drug-loaded SEMC (F2) and the F2-ERS was the highly stable macroporous material. A rapid adsorption transition in the P/P_0_ range of 0.99–1.0 for macroporous SEMC, F2, and the F2-ERS (Figure 3) suggested a unimodal surface pore size and the nonexistence of hysteresis, indicating the presence of macropores and the existence of mesoporosity (17.0 to 3000.0 Å) concerning the connectivity of the porous network due to the occurrence of unrestricted monolayer–multilayer adsorption at high P/P_0_ (1.0), which was also reported previously [55].

### 3.4. Fourier Transform Infrared (FTIR) Spectroscopy

**Figure 4:**
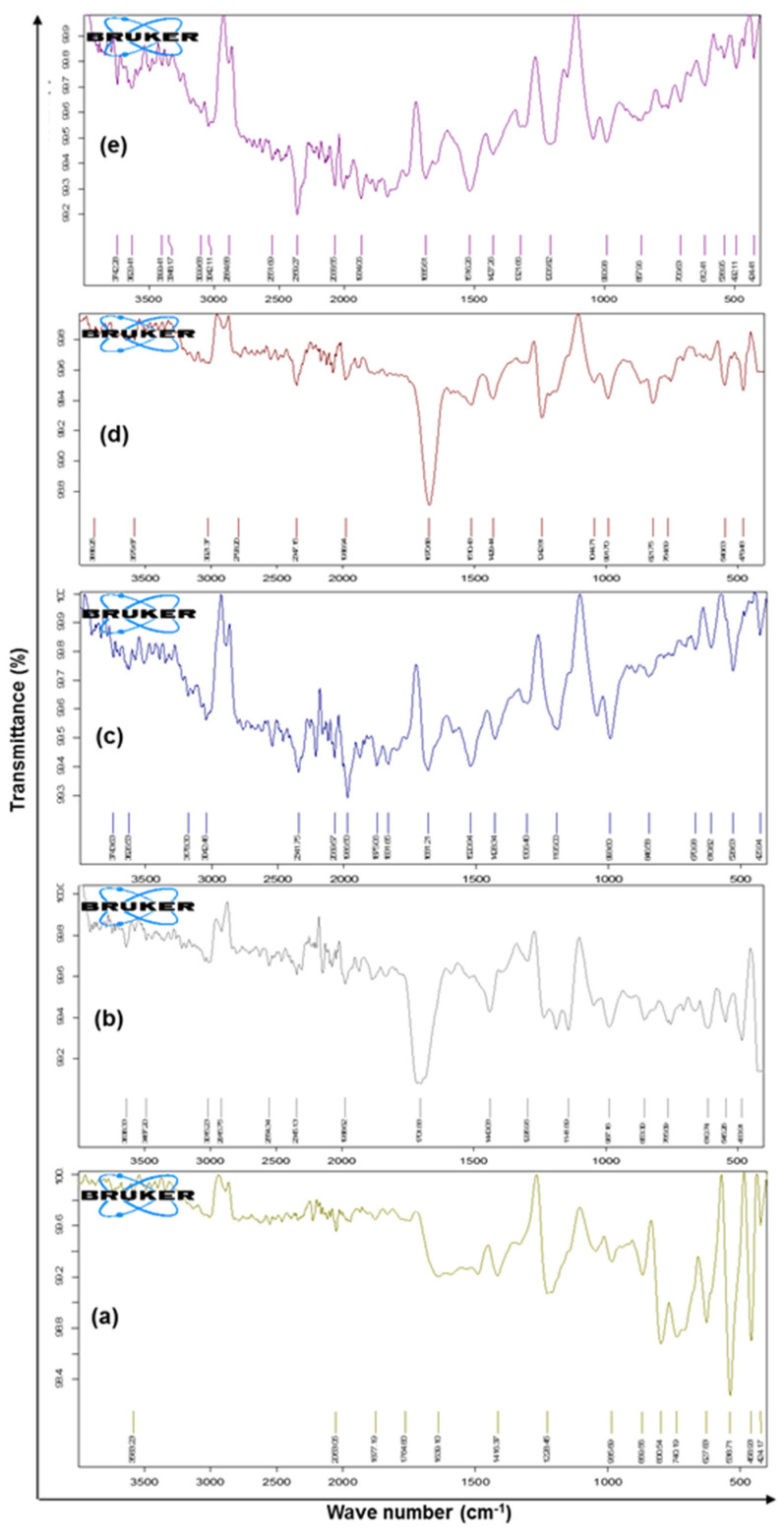
FTIR spectrum of pure drug 5-FU (**a**), Eudragit RS-100 (ERS) (**b**), SEMC alone (**c**), 5-FU-loaded SEMC (F2) (**d**), and 5-FU-loaded ERS-coated (F2-ERS) © (**e**).

The FTIR spectra of pure drug 5-FU, Eudragit RS-100, macroporous SEMC, drug-loaded SEMC (F2), and the F2-ERS were recorded in the range of 4000–400 cm^−1^ wavenumber by a Bruker Tensor-27 spectrophotometer (USA) using KBr pellets. The FTIR spectra bands at 1661, 1449, 3136, 1430, and 1246 cm^−1^ indicated the presence of C=O, C=C, N-H, C-F, and C-N stretching vibrations corresponding to 5-FU, while the peak at 1349.35 cm^−1^ refers to pyrimidine compound vibration, confirming 5-FU (Figure 4a) [56,57]). Eudragit RS 100 showed O-H stretching of the hydrate band at 3487.20 cm^−1^, C=O stretching of saturated aldehyde at 1701.88 cm^−1^, N-R stretching of quaternary amine salt at 1440.08 cm^−1^, and C-O-C stretching of a strong ester band at 11,411,296 cm^−1^, as shown in Figure 4b. [58]. The FTIR spectrum exhibits SEMC stretching: 3450–3550 cm^−1^ O-H stretch of -OH groups, 3042.46 cm^−1^ stretch (-CH_2_-), C-H stretch 2693 cm^−1^ (-CH_2_-), C-H (va) stretch 2840 cm^−1^ (-CH_2_-), C-H (va) stretch 2840 cm^−1^ (-CH_2_-), C-H (va) stretch 2840 cm^−1^, -CH_2_- (shoulder) C=O stretch of -CO_2_H 1650 cm^−1^, C=C stretch 1428–1520 cm^−1^, (-CH_2_-) 1196 cm^−1^, and (-C-O-C-) stretch 670–846 cm^−1^ (-CH_2_-) rocking (Figure 4c) [59,60]. Drug-loaded uncoated SEMC-FU (F2) displayed the matches of spectra of 5-FU and SEMC for various functional groups such as stretch 1670.68, 1510.48, 3021.37, 1429.44, and 1242.81 cm^−1^, indicating the presence of C=O, C=C, N-H, C-F, and C-N stretching vibrations corresponding to 5-FU 1342.21 cm^−1^, which refers to pyrimidine compound vibration (Figure 4d). The formulation F2-ERS displayed matches between the spectra of 5-FU, ERS, and SEMC that were found for different functional groups. Symmetric C-H stretching bands at 2884.88 cm^−1^, 3042.11 cm^−1^, and 1321.66 cm^−1^ for 5-FU, ERS stretching of the hydrate band at 3399.41 cm^−1^, C=O stretching at saturated aldehyde at 1685.61 cm^−1^, N-R stretching of quaternary amine salt at 1427.26 cm^−1^, and C-O-C stretching of a strong band of ester at 1141,1206 cm^−1^ and SEMC displaying stretching: 3348.17–3399.41 cm^−1^ O-H stretch of -OH groups, 3042.42 cm^−1^ (-CH_2_-) C-H (νa) stretch, 2884.88 cm^−1^ (-CH_2_-) C-H (νb) stretch, 2551.63 cm^−1^ (shoulder) O-H stretch of H bonded -CO_2_H, 1685.63 C=O stretch of -CO_2_H, 1650 cm^−1^ C=C stretch—1427–1516 cm^−1^ (-CH_2_-), 1196 cm^−1^ (-C-O-C-) stretch, and 706.63–857.96 cm^−1^ (-CH_2_-) rocking were observed. The comparable FTIR for the spectra of 5-FU, ERS, and SEMC was recorded (Figure 4e). From the studies, it could be concluded that 5-FU was compatible with the excipients used in the present study. However, the broadening and decrease in peak intensity were observed in the spectrum of F2-ERS, which indicated there was no chemical interaction between drug and the polymer.

### 3.5. Powdered X-ray Diffraction (PXRD)

**Figure 5:**
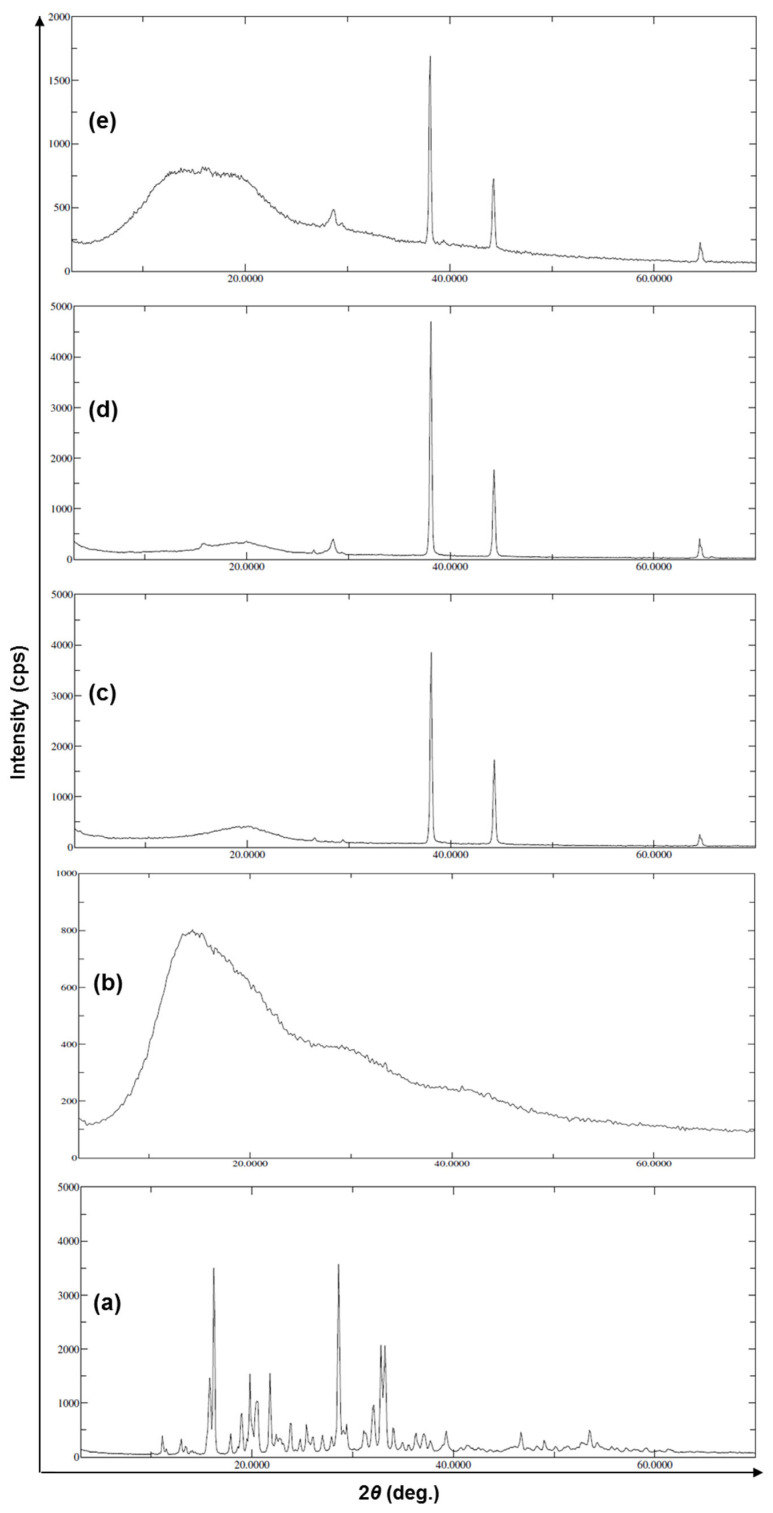
XRD patterns of pure drug 5-FU (**a**), Eudragit RS-100 (ERS) (**b**), SEMC alone (**c**), 5-FU-loaded SEMC (F2) (**d**), and 5-FU-loaded ERS-coated (F2-ERS) (**e**).

The XRD spectra of the samples in comparison to the pure drug (5-FU) were illustrated in Figure 5. The spectrum of 5-FU (Figure 5a) clearly showed the highest peak with an intensity of 2701 cps at the 2*θ* of 28.6 deg with a Bragg’s (d-value) of 3.11, and I/I_0_ was 100. In addition, the second highest peak with 2594 cps intensity at 2*θ* of 16.2 deg, with the d-value 5.46 and I/I_0_ of 97, which could define the crystallinity of the pure 5-FU. The presence of a broad peak with low intensity (44 cps) at 2*θ* of 16.2 deg with the d-value 5.82 and I/I_0_ of 83 in case of ERS (Figure 5b) indicated the amorphous nature of Eudragit RS-100. Similarly, two characteristic high-intensity peaks (3018 and 1394 cps) at 2*θ* (38.0 and 44.3 deg) with d-values (2.36 and 2.04) and I/I_0_ (100 and 44) that respectively appeared in case of SEMC alone (Figure 5c). When 5-FU was loaded in SEMC (F2), the characteristic crystalline peaks of 5-FU were almost disappeared, but the characteristic peaks of SEMC were obvious (Figure 5d), indicating that the 5-FU was loaded inside the pores of the SEMC in the amorphous form. In addition, the characteristic crystalline peaks of 5-FU were not seen after ERS coating on 5-FU-loaded SEMC (Figure 5e), but only the characteristic peaks of SEMC were found with low intensities (1193 and 484 cps) at 2*θ* (38.1 and 44.3 deg) with d-values (2.36 and 2.04) and I/I_0_ values of 100 and 41, respectively in F2-ERS. The above findings are in agreement with a previous report of 5-FU-loaded PCL and PLGA-NPs [61] as well as 5-FU-loaded chitosan-NPs [62,63]. Conclusively, the absence or disappearance of the characteristic crystalline peaks of 5-FU in F2 and F2-ERS indicate the existence of 5-FU in an amorphous state in the pores and matrix of the SEMC. Moreover, XRD analysis suggested that most of the 5-FU molecules were entrapped within the SEMC-matrix rather than adsorbed onto the surfaces of SEMC. These findings were further confirmed by the FTIR analysis (as mentioned in Section 3.4) of all five samples.

### 3.6. Differential Scanning Calorimetry

**Figure 6:**
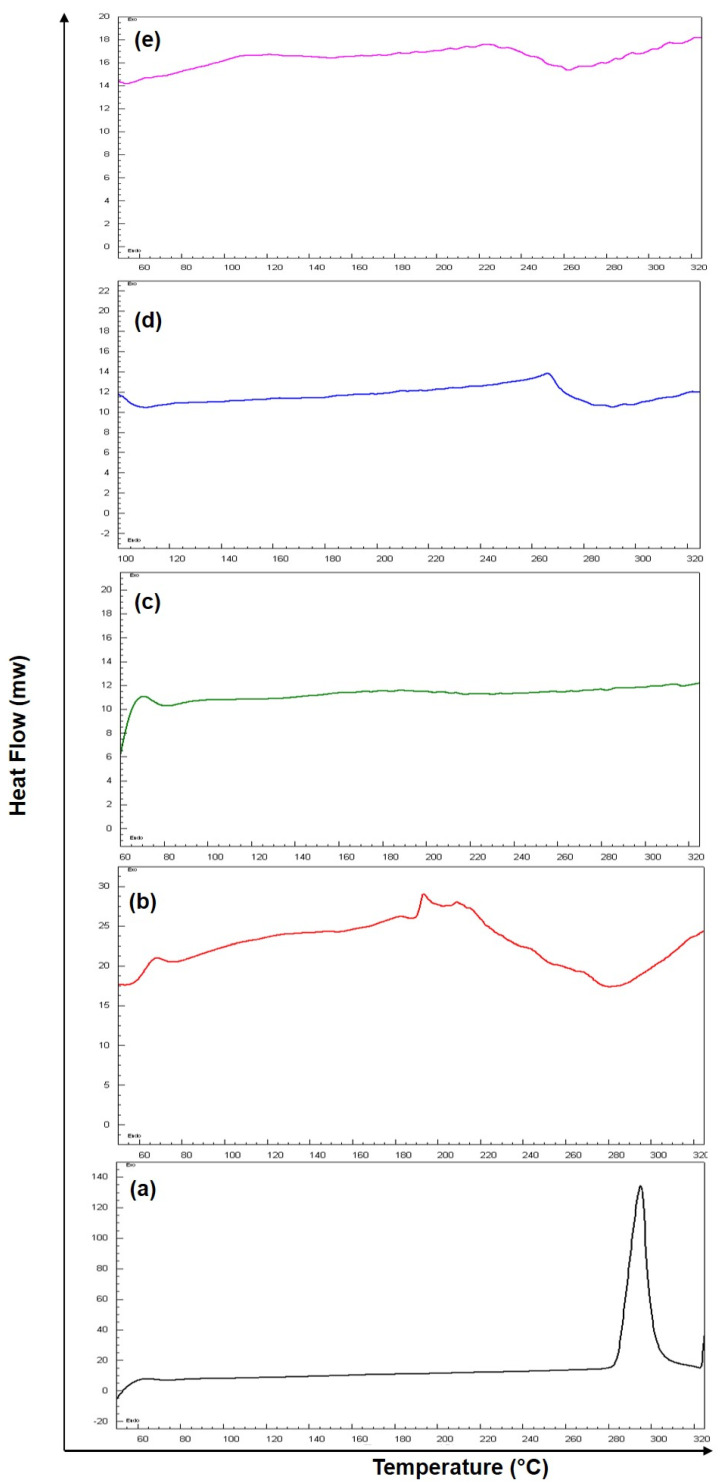
DSC thermogram of pure drug 5-FU (**a**), Eudragit RS-100 (ERS) (**b**), SEMC alone (**c**), 5-FU-loaded SEMC (F2) (**d**) and 5-FU-loaded ERS-coated SEMC (F2-ERS) (**e**).

The overlay DSC thermograms of pure 5-FU, Eudragit RS-100 (ERS), SEMC alone, 5-FU-loaded SEMC (F2), and 5-FU-loaded ERS-coated (F2-ERS) are presented in Figure 6. The DSC curve of 5-FU has shown a single endothermic peak at 286.5 °C (Figure 6a). The DSC thermogram of the pure 5-FU also showed a sharp melting endotherm peak at ≈286.9 °C followed by decomposition, which was in agreement with those reported previously [64], while the endothermic peak of pure ERS appeared around 193 °C (Figure 6b), and no specific peak was found in case of blank SEMC in the present investigation (Figure 6c). The 5-FU-loaded SEMC (F2) formulation exhibited an endothermic peak, although it was not sharp at around 272.5 °C (Figure 6d), suggesting that 5-FU was in amorphous form and the majority of the drug was adsorbed into the porous structure of the SEMC. Moreover, a slight decrease in the melting temperature for 5-FU was noted in case of F2, which might be attributed to the loss of crystallinity of the drug, whereas a shifted small broad endothermic peak at 200 to 260 °C suggested that the drug was either totally or partially converted into amorphous form and furthermore, no characteristic peak of 5-FU was observed. The reduction of height and sharpness of the endotherm peak may be due to the presence of polymers in the 5-FU-loaded SEMC (F2-ERS); the downward shift indicated the loss of mass (due to solvent evaporation, loss of moisture, and degradation) upon heating. This indicated that the adsorbed drug into the porous structure of the SEMC–matrix was further and well coated by Figure 6e. Conclusively, the DSC results of drug-loaded SEMC and its coating with ERS suggested that the 5-FU molecules were adsorbed in the porous exterior surfaces of the SEMC in an amorphous state. These results corroborate the previous studies [65,66].

### 3.7. Effect of 5-FU Concentration on Encapsulation and Its Loading into SEMC

To encapsulate 5-FU into SEMC, the solubility of 5-FU was enhanced up to 150 mg by dissolving the drug in each mL 1:1 (*v*/*v*) mixture of 1N NH_4_OH and ethyl alcohol, because the higher solubility of 5-FU accelerates the improved encapsulation and loading into SEMC because the encapsulation and loading of any drug into SEMC depends upon the solubility of the drug in a hydro-alcoholic or aqueous medium [22,49]. The vacuum-assisted method for encapsulation and loading enables the SEMC to encapsulate a high amount of drug into its channels and internal cavities, which might be due to the enforced passage of drugs and the elastic nature of SEMC surfaces as well as the physical and chemical features of the nano-sized channels and internal cavities of SEMC [20,33]. Based on the previous reports, the encapsulation of 5-FU greatly depends upon the ratios of drugs and carriers used for the development of controlled-release formulations [22]. Therefore, we tried to optimize the encapsulation and loading of 5-FU into SEMC by considering three different ratios of drug–SEMC to get maximum encapsulation and loading of 5-FU through the vacuum-assisted method. A direct method was applied for the determination of encapsulation efficiency (%EE) and drug-loading capacity (%DL). An optimum encapsulation and loading of 59.81% and 19.94%, respectively (in case of F2, *p* < 0.05) was found when 100 mg of 5-FU and 200 mg of SEMC was used, while it was lower (47.66% and 9.53%, respectively) at 50 mg of 5-FU and when 200 mg of SEMC was used (in case of F1). By increasing the amount of 5-FU (150 mg, in case of F3) further, there was no significant increase in the encapsulation (58.86% only) as compared to F2, while the drug loading was increased significantly (i.e., 25.53%). No significant improvement in %EE in the case of F3 indicated that the higher drug amount could increase the encapsulation efficiency of SEMC [67]. This was attributed to the fact that the encapsulation and loading greatly depend upon the physicochemical properties of the drug and carrier as well when the above-mentioned method was used to prepare the SEMC-based formulations [67,68]. An obvious improvement in %DL in the case of F3 (25.23%) was noted, which was due to the presence of the highest amount of 5-FU in F3 that influenced the calculation. It was contrary to the prior study of Alshehri et al., 2016 [13]. They reported a decreased loading (94.6 to 82.8%) of ibuprofen when the concentration of the drug was increased (50 to 400 mg/L), which might be attributed to the limited site availability for drug loading [13,69]. Based on the optimum encapsulation and drug loading as well as optimum size, F2 was chosen for further experiments and also only F2 was subjected to ERS coating. When the amount of 5-FU was highest (150 mg, in F3) among all, the %DL was highest (25.23%). Contrary to this, the %EE and %DL were 56.23% and 10.22%, respectively in the case of ERS-coated F2 formulation, which was due to the higher amount of total excipients (including 5 mL of 5% ERS) as compared to uncoated F2.

### 3.8. In Vitro Release of 5-FU

**Figure 7:**
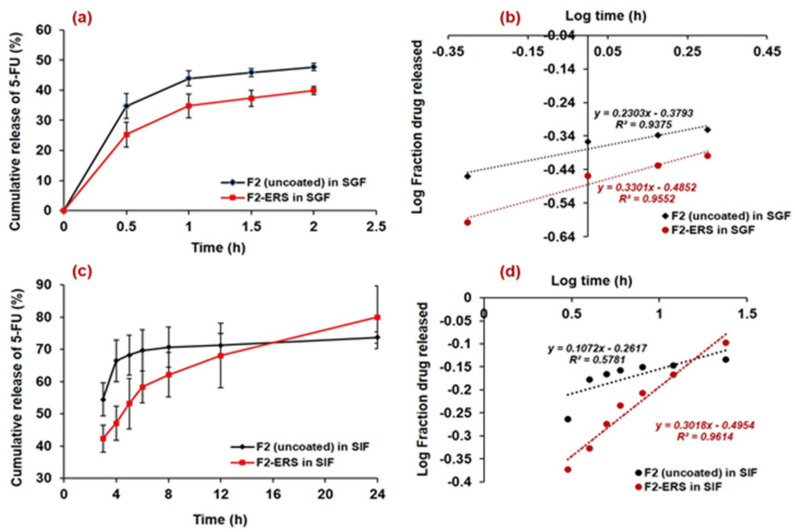
In vitro release profiles of 5-FU-loaded spores (uncoated and ERS-coated) in SGF (**a**); Korsmeyer–Peppas plots in SGF (**b**); release profiles of 5-FU-loaded spores (uncoated and ERS-coated) in SIF (**c**); and release kinetics model (Korsmeyer–Peppas) plots in SIF (**d**).

The in vitro drug release profiles of 5-FU loaded spores (uncoated (F2) and E-RS coated (F2-ERS)) in SGF (pH 1.2) and SIF (pH 6.8) is presented in Figure 7a and c, respectively. Around 34% of the drug was released within 0.5 h from F2 (uncoated spores) in the SGF release medium, while it was around 25% from the F2-ERS in the same release medium (SGF), and the cumulative amount of drug released at 2 h was noted around 47% and 39% from F2 and F2-ERS, respectively. Similarly, the higher release of 5-FU (around 54% from F2 and 42% from F2-ERS at 3 h) was observed in SIF release media. At 24 h, around 73.6% and 79.9% of 5-FU were released from F2 and F2-ERS, respectively in SIF. The prolonged-release pattern of 5-FU from F2-ERS was attributed to the Eudragit^®^ RS-100 coating. The ERS has quaternary ammonium groups in its structure, but it has pH-independent solubility and remains almost insoluble in aqueous media, but they are swellable and permeable [32]. The swelling behavior of ERS might be the reason for the higher drug released from the F2-ERS. Meanwhile, increased drug release from the uncoated spores might be attributed to the increased dissolution rate of the drug present on the surface of the spores as well as the rapid exit of the drug from the nano-channels present in the spore’s wall [48].

A prolonged and controlled release of 5-FU was observed from the F2-ERS in SIF up to 24 h, which might be attributed to the increased diffusion pathway and tortuosity of the spores due to the ERS coating [26]. The present delivery system comprised of 5-FU-encapsulated SEMC and its coating with ERS (pH-independent polymer) revealed its probability for the colonic delivery of 5-FU at 6.8 pH, which was well demonstrated by the successful sustained release of 5-FU until 24 h in SIF. The results obtained in the present study were also supported by the previous study conducted for the colonic delivery of 5-aminosalicylic acid for 12 h at 6.5 pH [70]. The release of 5-FU from the F2-ERS was found to be more sustained, which might be controlled due to the ERS coating on F2, and there was no lag time in the release of 5-FU, which might be associated with the pH-independent dissolution of Eudragit^®^ RS-100. The sustained release of 5-FU from F2-ERS was further substantiated by plotting the log time versus log fraction of 5-FU released (Korsmeyer–Peppas release model), as represented in Figure 7b. The regressed line of this plot generated the coefficient of correlation (R^2^) value of 0.961. From the slope of this curve, the diffusion exponent (*n*-value) was calculated and found to be 0.131. The *n*-value suggested that the mechanism of drug release principally followed the Fickian-diffusion type. A sustained but slightly higher 5-FU release (79.9% at 24 h) was found in the case of F2-ERS, which might be due to the polymer erosion in SIF. The release data obtained in 2 h study (in SGF) were also fitted into different kinetic models. The release of 5-FU from uncoated SEMC was higher (47.7% at 2 h) as compared to the ERS-coated SEMC in SGF. This was due to the acidic pH of SGF that could not properly solubilize the ERS coating at pH 1.2. The log time versus log fraction of 5-FU released (Korsmeyer–Peppas release model) is represented in Figure 7d. The regressed line of this plot generated the coefficient of correlation (R^2^) values 0.955 and 0.938 (for F2-ERS and F2 uncoated, respectively). From the slope of the curves, *n*-values (0.143 and 0.230) were obtained that suggested that the release of 5-FU primarily followed the Fickian-diffusion mechanism.

Thus, we could postulate that the release of 5-FU from F2-ERS was the combination of dissolution, diffusion, and polymer–erosion, which was similar to the previous reports [13,22,71]. The in vitro drug release data from F2-ERS has shown the prolonged release of 5-FU and could be controlled at pH conditions of GIT due to the polymer coating, which can be very much advantageous to treat the cancers of the colon, stomach, breast, etc. with reduced dosing frequency.

### 3.9. Stability of 5-FU Loaded SEMC (Uncoated and ERS-Coated)

Due to the excellent biocompatibility, low toxicity, consistency in size, resistance to even tough chemical conditions, and high-temperature stability, SEMC obtained from pollens of different species have been used as green carriers for many drugs [72]. There are numerous reports available regarding the stable shelf-life of pollens and their extracts [73,74,75]. Thus, in the present investigation, only a few parameters including the size, encapsulation (%EE), and drug-loading capacity (%DL) of SEMC were determined after storage of 5-FU-loaded uncoated and ERS-coated formulations. The measured values for size, %EE and %DL of F2 and F2-ERS at different time points are presented in Table 2. The results indicated no significant changes in the measured parameters (size, %EE, and %DL) at 30 ºC for 1 month. A significant (*p* < 0.05) change is assumed if the measured values show a 5% increase in size or decrease in %EE and %DL as compared to the initial (0 days) values of a batch [76]. A non-significant (*p* < 0.05) increase in the size was observed in the case of F2 and F2-ERS on the 15th day and 30th day (Table 2), which might be due to the moisture adsorption and swelling property of SEMC. A slight decrease in %EE and %DL of 5-FU was noticed in F2 and F2-ERS on the 15th and 30th day (3.04% and 4.79% in F2 and 2.18% and 2.87% in F2-ERS). Comparatively, the more reduced %EE and %DL in the case of uncoated SEMC might be due to the moisture adsorption phenomenon of uncoated SEMC. Meanwhile, the ERS coating hindered the moisture adsorption by SEMC in the case of F2-ERS; therefore, here, only a very small percentage of reduction in %EE and %DL were found. The almost negligible findings, especially the %EE and %DL, indicated that the entrapped drug was found to be stable at the mentioned storage temperature during the short-term stability testing for one month.

### 3.10. In Vivo Pharmacokinetics

**Figure 8:**
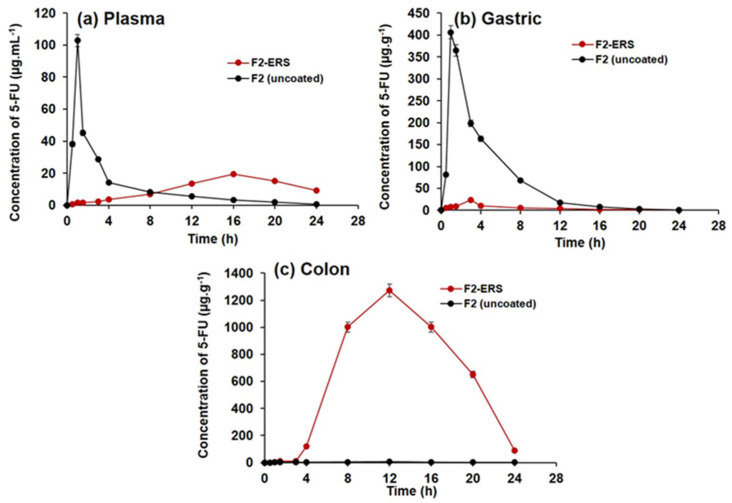
Concentrations versus time profiles of 5-FU in plasma (**a**); gastric tissue homogenates (**b**); and colonic tissue homogenates (**c**), after oral administration of 5-FU-loaded SEMC (F2 uncoated) and colon-directed Eudragit RS-100-coated F2. The data are represented as mean ± SD, (*n* = 3).

The efficiency of drug-loaded ERS-coated SEMC (F2-ERS) for colon-directed delivery of 5-FU was evaluated for pharmacokinetics and GI tract distributions in rats in comparison to the control treatment (F2). Equivalent amounts of the two formulations were dispersed in normal saline to achieve the dose of 5-FU (8.05 mg/kg b.wt). The concentration versus time profiles of 5-FU are presented in Figure 8a–c, and the pharmacokinetic parameters are summarized in Table 3. The oral administration of F2 displayed a rapid release of 5-FU in plasma (Figure 8a). The C_max_ of 5-FU was 102.82 ± 3.85 μg/mL after 1 h. Subsequently, the plasma concentration decreased quickly with a half-life (t_1/2_) of around 4.04 h, AUC_0-t_ was 264.1 ± 9.8 μg/mL.h, MRT_0-inf_ was 5.2 h, and the observed Cl/F was 0.03 (mg)/(μg/mL)/h. Most of the drugs become absorbed in the upper gastrointestinal tract; therefore, it seems that F2 was rapidly released in the gastric cavity [77]. In contrast, the observed C_max_ of 5-FU from F2-ERS (19.47  ±  0.61 μg/mL) was 80.45% lower than that of the F2 (102.82 ± 3.85 μg/mL). The T_max_ after the administration of F2 was 1 h, which was significantly different (*p*  <  0.05) from the T_max_ (16 h) obtained with F2-ERS (Table 3). This longer T_max_ indicated that the F2-ERS effectively prevented drug release in the upper part of the GI tract. The further pharmacokinetic parameters endorsed the slow release of drug as MRT0-inf, and the observed volume of distribution (V_z_/F) increased 269.14% from 5.19 h (for F2) to 20.57 h (for F2-ERS) and 41.17% from 0.18 (mg)/(μg/mL) for F2 to 0.24 (mg)/(μg/mL) for F2-ERS, respectively; however, the rate of clearance decreases from 0.03 to 0.02 ((mg)/(μg/mL)/h), which is 25.06% as compared to F2. The pharmacokinetics parameters indicated that the enzyme-mediated degradation of polysaccharides of SEMC and ERS in the colon was a slow process [78]. The pharmacokinetic data have shown that the MRT of F2-ERS was longer than that of the F2. The prolonged MRT of the drug in the colon was supplemented with sustained drug release property of the F2-ERS at the target site, which was noticed from the plasma concentration profile of 5-FU. These results were in agreement with the previous report by Wei He et al. 2008 [79]. The colon, a homogeneous reservoir, elicits slow and constant release into systemic circulation similar to continuous infusion, which is highly recommended in the chemotherapy of cancer patients with a short half-life 5-FU [46,80]. The elimination (K_el_) of F2-ERS was about 47.05% more reduced than that of the F2, displaying slower clearance of the drug from the body, which might be due to the encapsulation of the drug inside the cages of the carrier (SEMC). The slow and decreased clearance of encapsulated drug increased the half-life (T_1/2_) by 83.12% from 0.09 to 0.17 per hour as compared to F2 uncoated. The relative bioavailability of the 5-FU from F2-ERS was found to increase by 4.35% as compared to F2. These results were in agreement with the previous report [46,77,79,81].

### 3.11. Tissue Distribution of 5-FU in Stomach and Small Intestine

The distribution of 5-FU into the stomach and small intestine after the administration of colon-directed F2-ERS and F2 is represented in Figure 5b. The mean peak concentrations of 5-FU in the tissues of the stomach and small intestine from F2 were 406.2 ± 15.04 μg/g at 1 h, 198.6 ± 6.9 μg/g at 3 h, 163.5 ± 5.6 μg/g at 4 h, and 16.9 ± 1.7 μg/g at 12 h. These data indicated that F2 was an immediate release formulation where a large quantity of 5-FU gets released in the upper part of the GI tract and small intestine. The pharmacokinetic data of gastric tissue and small intestine of F2-ERS exhibit a reduction in Cmax (μg/mL), AUC0-t (μg/mL*h), and Ke (1/h) that is 94.20%, 92.96%, and 785.71%, respectively as compared to the F2 uncoated formulation and increased in half-life (t_1/2_), Tmax (h), MRT0-inf (h), Vz/F (mg)/(μg/mL), and Cl/F {(mg)/(μg/mL)/h} that is 352.71%, 200%, 142.14%, 5732.52%, and 1140%m respectively as compared to the F2 (uncoated) formulation indicating that a negligible amount of 5-FU was released in gastric tissues and small intestine from the colon-directed F2-ERS and that the ERS coating remained intact during the transit of the SEMC through the stomach and small intestine. A sharp decrease in the concentration of 5-FU was found with F2, which might be attributed to the processes of absorption, systemic distribution, and further movement toward the region of the small intestine.

### 3.12. Tissue Distribution of 5 FU in Colon Tissues

The distribution of 5-FU into the colon tissues after administration of the F2 and F2-ERS are represented in Figure 5c. Significant differences in 5-FU concentrations in colon tissues were observed after administration of the two formulations (*p* < 0.005). Pharmacokinetic parameters of F2-ERS display a significant drastic increase in Cmax (μg/mL), AUC0-t (μg/mL*h), and Ke (1/h), that is 26,896.39%, 24,890.82%, and 200% respectively and decreased in half-life (t_1/2_), MRT0-inf (h), Vz/F (mg)/(μg/mL), and Cl/F {(mg)/(μg/mL)/h} that is 65.97%, 12.15%, 99.79%, and 99.01%, respectively as compared to F2 (uncoated) formulation. The maximum 5-FU concentration (C_max_) from F2-ERS in colon tissues was 1271.5 ± 47.09 μg/g at 12 h, following 1001.5 ± 37.09 μg/g at 16 h, 650.4 ± 24.08 μg/g at 20 h, and 90.4 ± 3.34 μg/g at 24 h, respectively. Meanwhile, F2 releases a negligible amount of 5-FU i.e., 4.7 ± 0.06 μg/g at 12 h, 2.55 ± 0.04 μg/g at 16 h, and 1.46 ± 0.02 μg/g at 24 h, respectively. A higher 5-FU concentration was achieved with the F2-ERS at all time points, and its relative bioavailability was 249.9 times more in colon tissues as compared to F2. The high concentration of 5-FU in the colon could be attributed to the protection of the SEMC from the environment of the stomach and small intestine due to ERS coating, thereby preventing drug release in the upper part of the GI tract. These results are in agreement with previous reports [46,77,78]. The recommended dose of 5-FU for colorectal cancer patients with adequate hematopoietic function and the average weight is 12 mg/kg intravenously once daily for 4 consecutive days. If there is no toxicity, the dosage is reduced to 6 mg/kg on days 6, 8, 10, and 12, after which therapy is discontinued. Intravenous administration (10–15 mg/kg per week as a single dose) continues maintenance therapy by either repeating the first course every 30 days after the last day of the previous course of therapy or when toxic signs arising from the initial course of treatment have subsided. This could take nine to 45 therapy courses over 12 to 60 months [82]. The daily intravenous dose is 500 mg/m^2^ body surface area as per this dose regimen, the adult dose 5-FU to be administered intravenously is 600 mg (mean body surface area = 1.2 m^2^). Thus, the dose of 5-FU to be administered specifically to the human colon would be 100 mg as per the mean surface area of the human colon (0.20 m2) [83]. Therefore, targeting the delivery of 5-FU to the colon reduces the dose and therefore the side effects of 5-FU therapy.

## 4. Conclusions

The present investigation concludes that the ERS-coated 5-FU-loaded SEMC can be explored for the effective colon-specific delivery of 5-FU. Moreover, the release of the encapsulated drug from the SEMC was found to be available for a longer duration. The pharmacokinetics and organ distribution studies revealed that the drug concentration was found higher in the colon tissue, with a little systemic exposure to the drug. The proposed system could be able to reduce the side effects of 5-FU due to its absorption from the upper part of the GI tract.

## Figures and Tables

**Table 1:** Formulation, encapsulation, and loading of 5-FU into SEMC.

Formulations (Ratio of 5-FU/SEMC)	Amount (mg)		%EE (Mean ± SD)	%DL (Mean ± SD)	Size (µ) (Mean ± SD)
	5-FU	SEMC			
F1 (1:4)	50	200	47.66 ± 2.34	9.53 ± 0.46	12.42 ± 2.94
F2 (1:2)	100	200	59.81 ± 4.19	19.94 ± 1.41	13.68 ± 3.91
F3 (3:4)	150	200	58.86 ± 4.04	25.23 ± 1.73	17.02 ± 2.94
F2-ERS coated (with 5 mL of 5% ERS)	100	200	56.23 ± 5.48	10.22 ± 0.99	15.47 ± 3.68

5-FU (5-Fluorouracil) and SEMC = Sporopollenin exine microcapsules.

**Table 2:** Time-dependent evaluation of size, encapsulation efficiency, and drug-loading capacity of F2 and F2-ERS stored at 30 °C for 30 days.

Stipulated Time Points	F2 Uncoated (Mean ± SD)			F2-ERS (Mean ± SD)		
	Size (µ)	%EE	%DL	Size (µ)	%EE	%DL
Initially	13.68 ± 3.91	59.81 ± 4.19	19.94 ± 1.39	15.47 ± 3.68	56.23 ± 5.48	10.22 ± 0.99
At 15th day	13.86 ± 3.85	58.04 ± 3.48	19.34 ± 1.16	15.72 ± 3.75	55.03 ± 5.04	10.01 ± 0.92
At 30th day	13.98 ± 3.81	56.95 ± 3.05	18.98 ± 1.02	15.87 ± 3.78	54.61 ± 5.24	9.93 ± 0.95

**Table 3:** Pharmacokinetic parameters of 5-FU from drug-loaded SEMC (F2-uncoated) and Eudragit RS-100-coated F2 (F2-ERS). Data were represented as mean SD, *n* = 3. All values represent mean ± SD. ^$^ *p* < 0.05 (F2-Uncoated); ANOVA, followed by Dunnett’s test.

Pharmacokinetic Parameters	Plasma			Colon			Gastric		
	F2-ERS ^$^	F2 (Uncoated) ^$^	Change in % Control	F2-ERS ^$^	F2 (Uncoated) ^$^	Change in % Control	F2-ERS ^$^	F2 (Uncoated) ^$^	Change in % Control
	Mean ± SD, *n* = 3	Mean ± SD, *n* = 3		Mean ± SD, *n* = 3	Mean ± SD, *n* = 3		Mean ± SD, *n* = 3	Mean ± SD, *n* = 3	
Ke (1/h)	0.09 ± 0.002	0.17 ± 0.0009	47.06	0.30 ± 0.0001	0.10 ± 0.000	−200.00	0.06 ± 0.000	0.28 ± 0.028	785.71
t_1/2_ (h)	7.38 ± 0.1875	4.03 ± 0.02	−83.13	2.31 ± 0.0001	6.79 ± 0.0001	65.98	11.04 ± 0.0001	2.43 ± 0.23	−354.32
T_max_ (h)	16.0 ± 0.0	1.0 ± 0.0	−1500.00	12.0 ± 0.0	12.0 ± 0.0	0.00	3.0 ± 0.0	1.0 ± 0.0	−200.00
C_max_ (μg/mL)	19.48 ± 0.61	102.82 ± 3.84	80.45	1271.53 ± 47.09	4.71 ± 0.06	−26,896.39	23.55 ± 0.41	406.23 ± 15.04	94.20
AUC_0-t_ (μg/mL·h)	252.60 ± 6.24	264.09 ± 9.84	4.35	16,209.05 ± 600.34	64.86 ± 0.91	−24,890.83	116.05 ± 2.01	1649.27 ± 71.14	92.96
AUC_0-inf_ (μg/mL·h)	350.54 ± 12.14	267.19 ± 9.83	−31.20	16,509.59 ± 611.46	79.16 ± 1.11	−20,755.98	129.98 ± 2.25	1651.17 ± 71.48	92.13
AUMC_0-inf_ (μg/mL·h^2^)	7212.19 ± 337.23	1387.17 ± 47.14	−419.92	223,020.8 ± 8260.02	1218.264 ± 17.16	−18,206.44	1322.44 ± 22.92	6941.41 ± 445.61	80.95
MRT_0-inf_ (h)	20.56 ± 0.29	5.19 ± 0.02	−296.15	13.51 ± 0.001	15.38 ± 0.001	12.16	10.17 ± 0.001	4.20 ± 0.09	−142.14
Vz/F {(mg)/(μg/mL)}	0.24 ± 0.005	0.17 ± 0.007	−41.18	0.002 ± 0.001	0.99 ± 0.01	99.80	0.99 ± 0.02	0.017 ± 0.001	−5723.53
Cl/F {(mg)/(μg/mL)/h}	0.023 ± 0.001	0.031 ± 0.001	25.81	0.001 ± 0.0001	0.102 ± 0.001	99.02	0.062 ± 0.001	0.005 ± 0.001	−1140.00

## Data Availability

The data generated from the experiments have been presented in the results.
